# Magnetic field observations in CoFeB/Ta layers with 0.67-nm resolution by electron holography

**DOI:** 10.1038/s41598-017-16519-7

**Published:** 2017-12-05

**Authors:** Toshiaki Tanigaki, Tetsuya Akashi, Akira Sugawara, Katsuya Miura, Jun Hayakawa, Kodai Niitsu, Takeshi Sato, Xiuzhen Yu, Yasuhide Tomioka, Ken Harada, Daisuke Shindo, Yoshinori Tokura, Hiroyuki Shinada

**Affiliations:** 1 Research & Development Group, Hitachi, Ltd., Hatoyama, 350-0395 Japan; 2 RIKEN Center for Emergent Matter Science (CEMS), Wako, 351-0198 Japan; 3 Hitachi High-Technologies Corporation, Hitachinaka, 312-0057 Japan; 4 National Institute of Advanced Industrial Science and Technology (AIST), Tsukuba, 305-8568 Japan; 5 Institute of Multidisciplinary Research for Advanced Materials, Tohoku University, Sendai, 980-8577 Japan; 6 Department of Applied Physics and Quantum-Phase Electron Center (QPEC), The University of Tokyo, Tokyo, 113-8656 Japan

## Abstract

Nanometre-scale magnetic field distributions in materials such as those at oxide interfaces, in thin layers of spintronics devices, and at boundaries in magnets have become important research targets in materials science and applied physics. Electron holography has advantages in nanometric magnetic field observations, and the realization of aberration correctors has improved its spatial resolution. Here we show the subnanometre magnetic field observations inside a sample at 0.67-nm resolution achieved by an aberration-corrected 1.2-MV holography electron microscope with a pulse magnetization system. A magnetization reduction due to intermixing in a CoFeB/Ta multilayer is analyzed by observing magnetic field and electrostatic potential distributions simultaneously. Our results demonstrate that high-voltage electron holography can be widely applied to pin-point magnetization analysis with structural and composition information in physics, chemistry, and materials science.

## Introduction

Innovation of new materials and devices requires structural and electromagnetic field observation in the research. Although atomic structure has become directly observable by high-resolution microscopy, nanometre-scale magnetic field distributions can be observed only at surfaces or in high magnetic fields. Since nanometre-scale magnetic field distributions in materials such as those at oxide interfaces^1^, in thin layers of spintronics devices^2–4^, in superlattice with magnetic and superconducting properties^5^, and at boundaries in magnets^6,7^ have become important research targets in materials science and applied physics, microscopy observing them at high resolution in magnetic-field-free conditions has been demanded. Among the various magnetic imaging techniques (such as Kerr microscopy, spin-polarized low-energy electron microscopy, scanning electron microscopy with spin polarization analysis, x-ray magnetic circular dichroism photoelectron emission microscopy, scanning transmission x-ray microscopy, magnetic force microscopy, and spin-polarized scanning tunneling microscopy)^8^, electron holography^9,10^ is one of the most powerful tools for obtaining quantitative values of local magnetic fields inside and outside a sample with high resolution.

The resolution of electron holography is determined by the spatial resolution of the transmission electron microscope (TEM) and the holography procedures used. To observe electrostatic potential distributions like those associated with atomic arrangements in crystals, we can place the sample in a magnetic field of about 2.4 MA/m in the gap of the objective lens. To observe the magnetic fields in and around the sample, however, we have to place the sample in a magnetic-field-free^11^ or magnetic-field-controlled^12^ position to maintain the inherent magnetic structure in the sample. The aberrations of the objective lens in these setups are large, limiting the spatial resolution. Placing the objective lens near the sample to suppress the aberrations has resulted in a spatial resolution of 0.77 nm^13^. Another effective way to reduce the aberrations is to use an aberration corrector, which for the last few decades has been developed and used to improve the resolution in electron microscopy^14–16^. A resolution of 0.5 nm was achieved for a sample located in a field-free position by using 300-kV TEMs with aberration correctors^17,18^. Recently, a 1.2-MV holography electron microscope with a spherical aberration corrector has been developed^19^, and a spatial resolution of 0.24 nm has been recorded for a sample located at a field-free position^20^.

**Figure 1:**
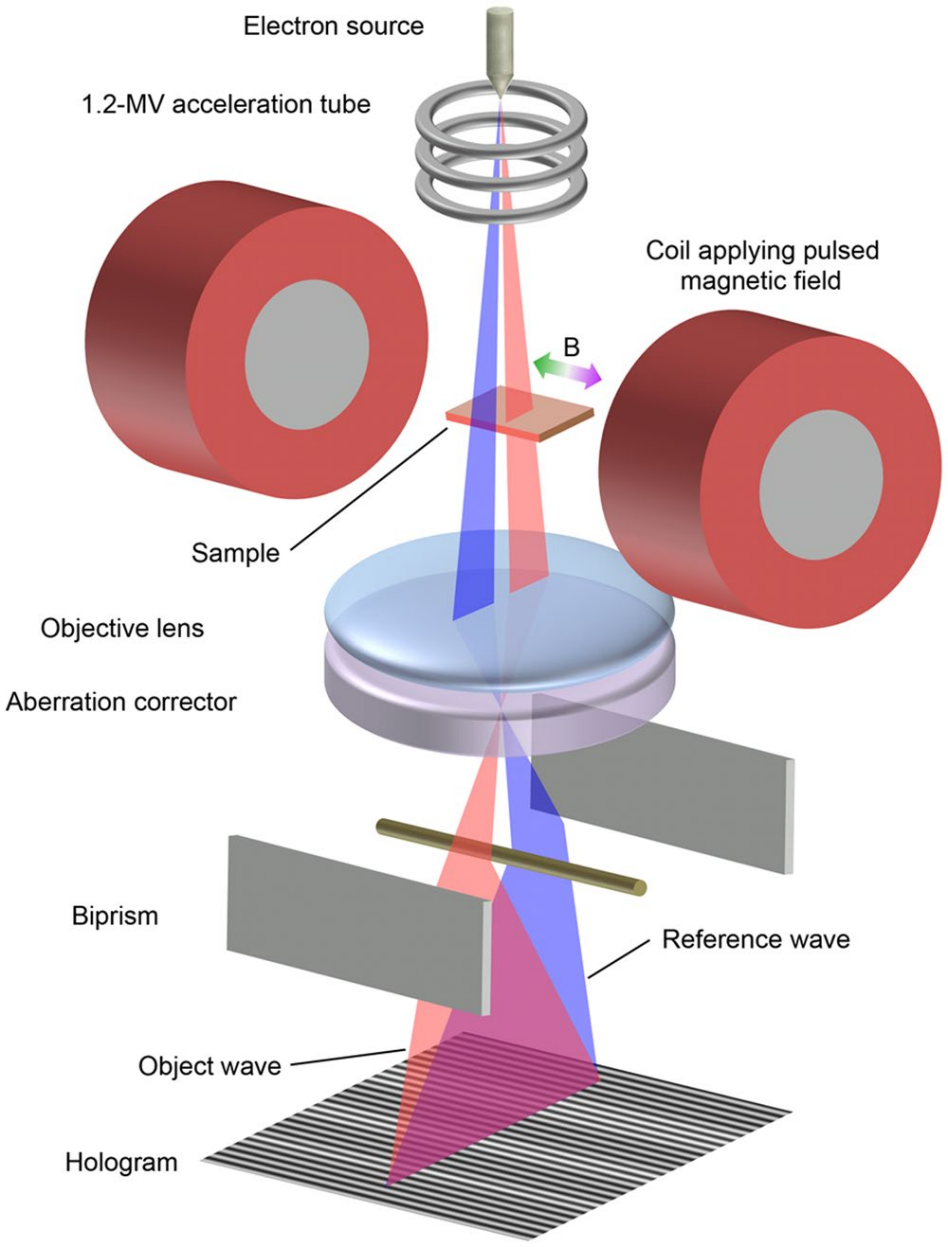
Experimental setup for high-resolution magnetic field observations. Magnetization direction in sample is reversed using pulsed magnetic fields generated by coils placed near two sides of the sample holder in an aberration-corrected 1.2-MV holography electron microscope. Holograms are formed by using a biprism to overlap object and reference waves.

Although the spatial resolution of TEMs has reached the subnanometre scale, the magnetic field observations made using an aberration-corrected holography electron microscope have so far been limited to 1-nm resolution^21^. Subnanometre resolution has not been achieved because there are additional difficulties: improving magnetic phase sensitivity and separating the electrostatic and magnetic phases at high spatial resolution. Here we show that the highest spatial resolution in magnetic field observations was achieved by developing a pulse magnetization system for use with the aberration-corrected 1.2-MV holography electron microscope (Fig. 1). The advantages of using a high-voltage electron microscope are (1) that, while a higher-energy electron wave is less sensitive to the electrostatic phase, the magnetic phase is highlighted because it is independent of the energy of the electron wave and (2) that the high penetration power of high-energy electrons allows us to integrate the magnetic phase for thick samples with less reduction of the resolution. The disadvantage is the possibility of electron irradiation damage.

## Results

### High-precision observation of the magnetic phase using a pulse magnetization system

The electrostatic potential and magnetic field are analyzed by using the phase shift *φ* of an electron wave, which is reconstructed from the interference pattern (hologram) between a reference wave and object wave^9,10^. The phase shift *φ* of an electron wave passing through a sample is given by

1 $$\phi (x,y)={C}_{E}\int V(x,y,z)dz-\frac{e}{\hslash }\int \overrightarrow{A}(x,y,z)d\overrightarrow{z}$$

where *C*_*E*_ is an interaction constant, *V*(*x*, *y*, *z*) is the electrostatic potential, and $\overrightarrow{A}(x,y,z)$ is the vector potential. The first term on the right is the electrostatic phase *φ*_*E*_, and the second term is the magnetic phase *φ*_*M*_. In a thin sample of thickness *t* with an *x*-directional magnetic field and negligible stray field, an in-plane component of the magnetic field *B*_*x*_ is given by

2 $${B}_{x}=\frac{\hslash }{et}\frac{\partial {\phi }_{M}}{\partial y}.$$

To separate the electrostatic and magnetic phases, a pulse magnetization system (Supplementary Information) was developed to reverse the magnetization in the sample without changing the geometrical configuration of the sample holder and stage. This can result in sample drifts significantly smaller than those occurring with conventional separation methods, e.g., flipping the sample and reversing the sign of the magnetic phase^22^, tilting the sample in the magnetic field and thereby reversing the magnetization in the sample^23^, and changing the temperature across the Curie temperature^24^. The electrostatic and magnetic phases could also be separated by using different acceleration voltages^25^, but the resolution would be limited by the lower voltage. The phases obtained in opposite magnetization states have different sign in the magnetic phase while having the same sign in the electrostatic phase. Subtracting these phases enables us to see the difference in the magnetic phases (the second term of equation (1)) due to differences between the in-plane magnetic field distributions before and after the magnetization reversal. Note that this idea can also be applied to other techniques for magnetic field observation using electron waves.

This procedure just requires a set of ending states produced by reversing the magnetization. The maximum pulsed magnetic field of 415 kA/m is larger than those of less 72 kA/m in the previously developed magnetization systems placed on^26,27^ and around^12,28^ the sample holder, which were designed to perform *in-situ* observation of the magnetic behaviour in a steady applied magnetic field. The developed system can be widely used to reverse the magnetization in hard magnets or magnets with a large shape effect, as demonstrated in a thin oxide magnet by applying a 207-kA/m pulsed magnetic field (Supplementary Information).

Another important factor for high-resolution magnetic field observation is the phase resolution. It is determined by the number of electrons that contribute to the pixels in the phase image reconstructed from the hologram^29^. To detect a small phase shift on a subnanometre scale, multiple sets of the holograms are acquired automatically and reconstructed phases are averaged out. The slight changes of the microscope alignments due to the pulse magnetic fields are corrected automatically by using preset alignment data for each condition. In a thin oxide magnet with uniform magnetization, the phase noise has been suppressed to ±0.0021 (2π/2990) rad (Supplementary Information), which is of the same order as the phase resolution of 2π/1000 rad deduced by setting the signal-to-noise ratio to 3.

### High-resolution magnetic field observations of CoFeB multilayer

**Figure 2:**
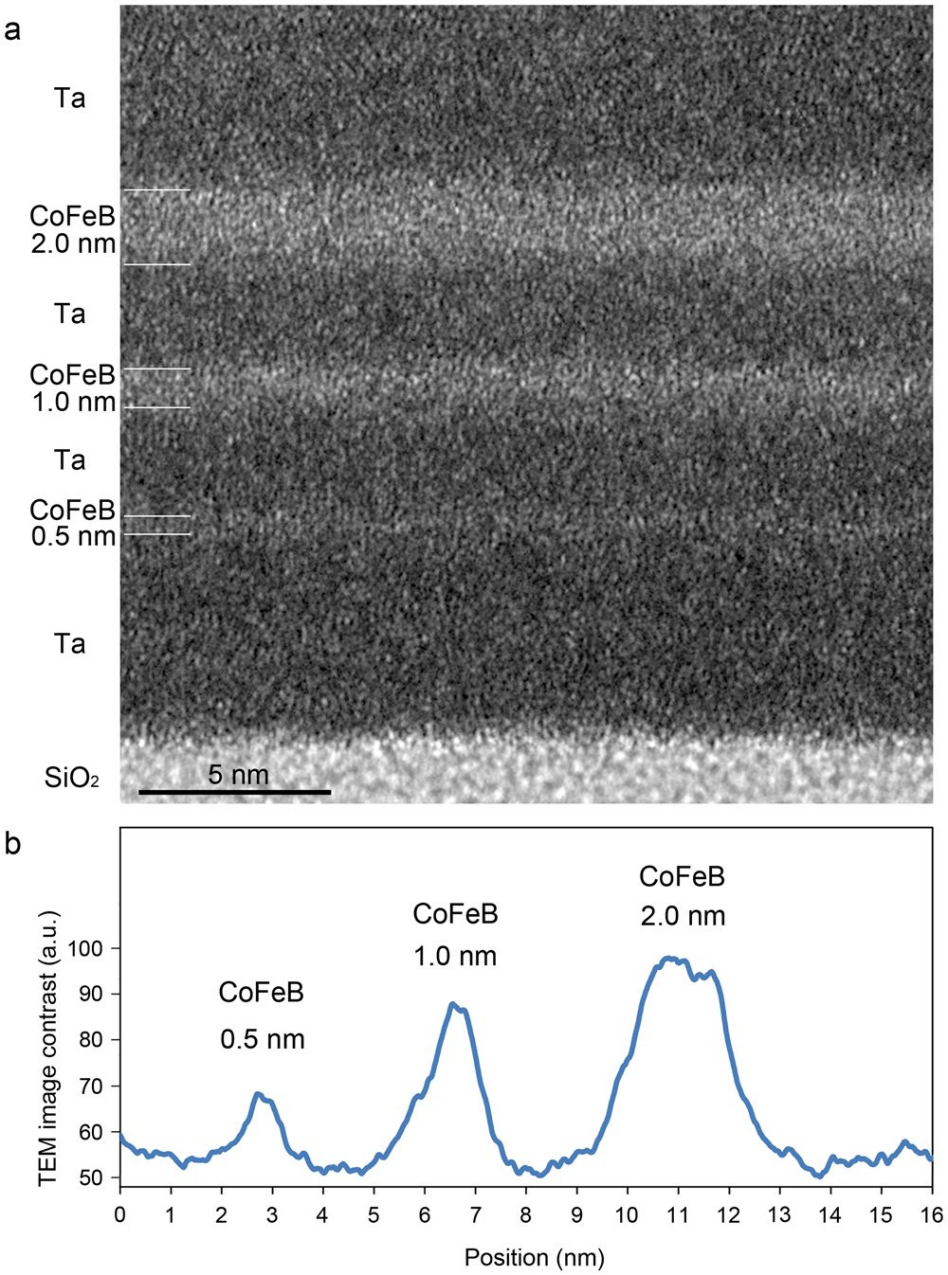
Structure of the CoFeB/Ta multilayer. (**a**) Transmission electron microscopic (TEM) image. (**b**) Profile of the TEM image contrast across the CoFeB/Ta multilayer shows gradual changes due to intermixing at the interfaces.

To demonstrate subnanometre-resolution magnetic field observation, a magnetic multilayer consisting of Ta(5.0 nm)/CoFeB(0.5 nm)/Ta(3.0 nm)/CoFeB(1.0 nm)/Ta(3.0 nm)/CoFeB(2.0 nm)/Ta(6.0 nm) was analyzed by applying pulsed magnetic fields parallel to the layers. Although the sample did not have a CoFeB/MgO interface, which plays a role for introducing perpendicular magnetization^2–4^, the robustness of ferromagnetism in CoFeB layers of different thickness is fundamentally important for manipulating the emergent phenomena in ferromagnetic/heavy-metal systems, such as the giant spin-Hall effect^3^ and Dzyaloshinskii-Moriya interaction^4^. We used the Fe-rich Co_20_Fe_60_B_20_ as the CoFeB magnetic layer. TEM observation revealed that the structure of the multilayer is amorphous and has intermixing^30^ at the CoFeB/Ta interfaces (Fig. 2). In the TEM image there is absorption-diffraction contrast indicating the elemental distributions and there is phase contrast reflecting the lattice information. Note that each Ta layer includes some nanocrystalline Ta.

**Figure 3:**
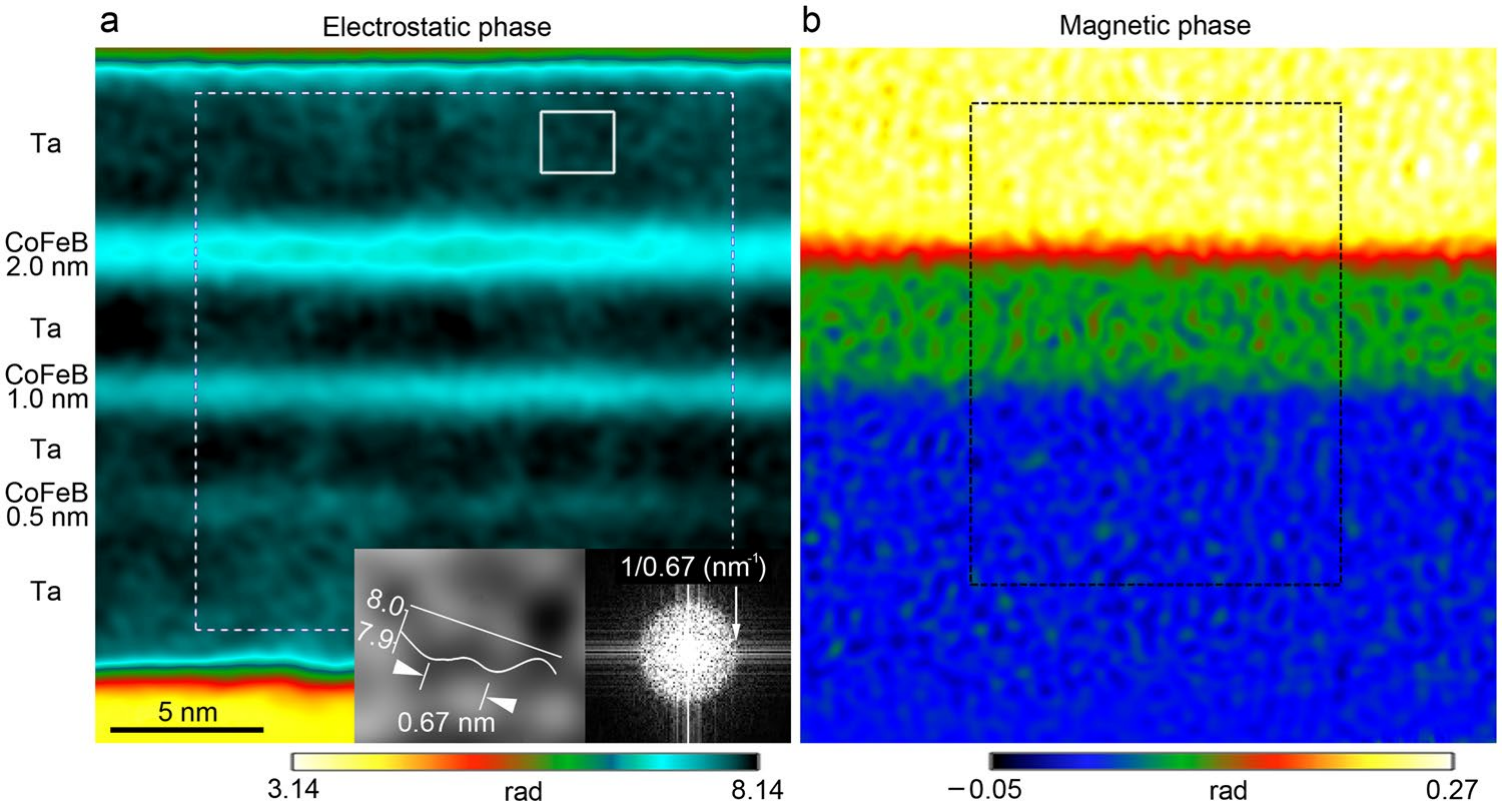
Obtained phases of CoFeB/Ta multilayer by electron holography with pulse magnetization system. (**a**) Electrostatic and (**b**) magnetic phases of CoFeB multilayer. Enlarged image of area indicated by white rectangle is inserted in the bottom of (**a**). Inset in the right bottom of (**a**) shows Fourier transform pattern of area indicated by white dashed square. Phase profiles of black dashed area are shown in Fig. 5.

**Figure 4:**
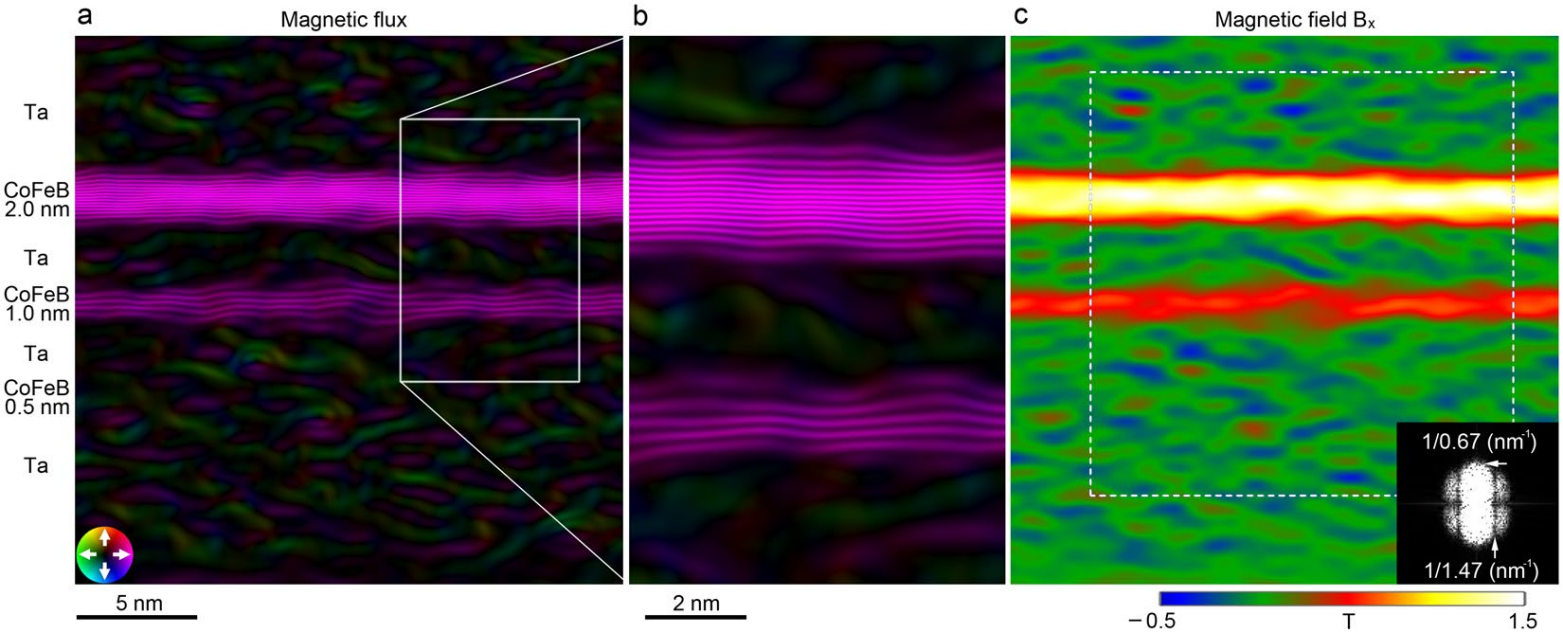
Magnetic flux and magnetic field of CoFeB/Ta multilayer. (**a**) Magnetic flux displayed by cosine of phase *φ*_M_ amplified 600 times (cos600*φ*_M_) with smoothing over the length scale of 1.43 nm parallel to the CoFeB layer. Constant flux of *h*/600*e* flows between adjacent contour lines. (**b**) Enlarged image of area indicated by white rectangle in the right of (**a**). (**c**) Magnetic field of *x*-directional component. Inset in (**c**) shows Fourier transform pattern of area indicated by the white-dashed square.

In the electrostatic phase (Fig. 3a), the individual CoFeB layers are clearly observed, even the 0.5-nm-thick one. To evaluate the spatial resolution of the phase image, the fast Fourier transform (FFT) pattern of the white dashed rectangular area is inserted at the bottom right in Fig. 3a. A spatial frequency of 1/0.67 nm^−1^ is confirmed in all directions in the FFT pattern. A structure with 0.67-nm separation is observed in the Ta layer, as indicated by the white arrows in Fig. 3a. These results show that the spatial resolution of the phase image is not degraded by the alignment and phase-decomposition processes. Since these hologram data processes are performed in the same way for the electrostatic and magnetic phases, the spatial resolution of the magnetic phase (Fig. 3b) is also 0.67 nm. Figure 4a and b show the in-plane magnetic flux distributions displayed by cosine of phase *φ*_M_ amplified 600 times (cos600*φ*_M_) with smoothing over the length scale of 1.43 nm parallel to the CoFeB layer. The in-plane magnetic fluxes are evident in the 2.0- and 1.0-nm-thick CoFeB layers but are not discernible in the 0.5-nm-thick layer. Figure 4c shows the *x*-directional component of the magnetic field. The magnetic field noise due to the shot noise in the original holograms in Fig. 4c is 0.10 T (standard deviation in Ta area). The detectable magnetic field in Fig. 4c is made 0.30 T by setting the signal-to-noise ratio to 3.

## Discussion

**Figure 5:**
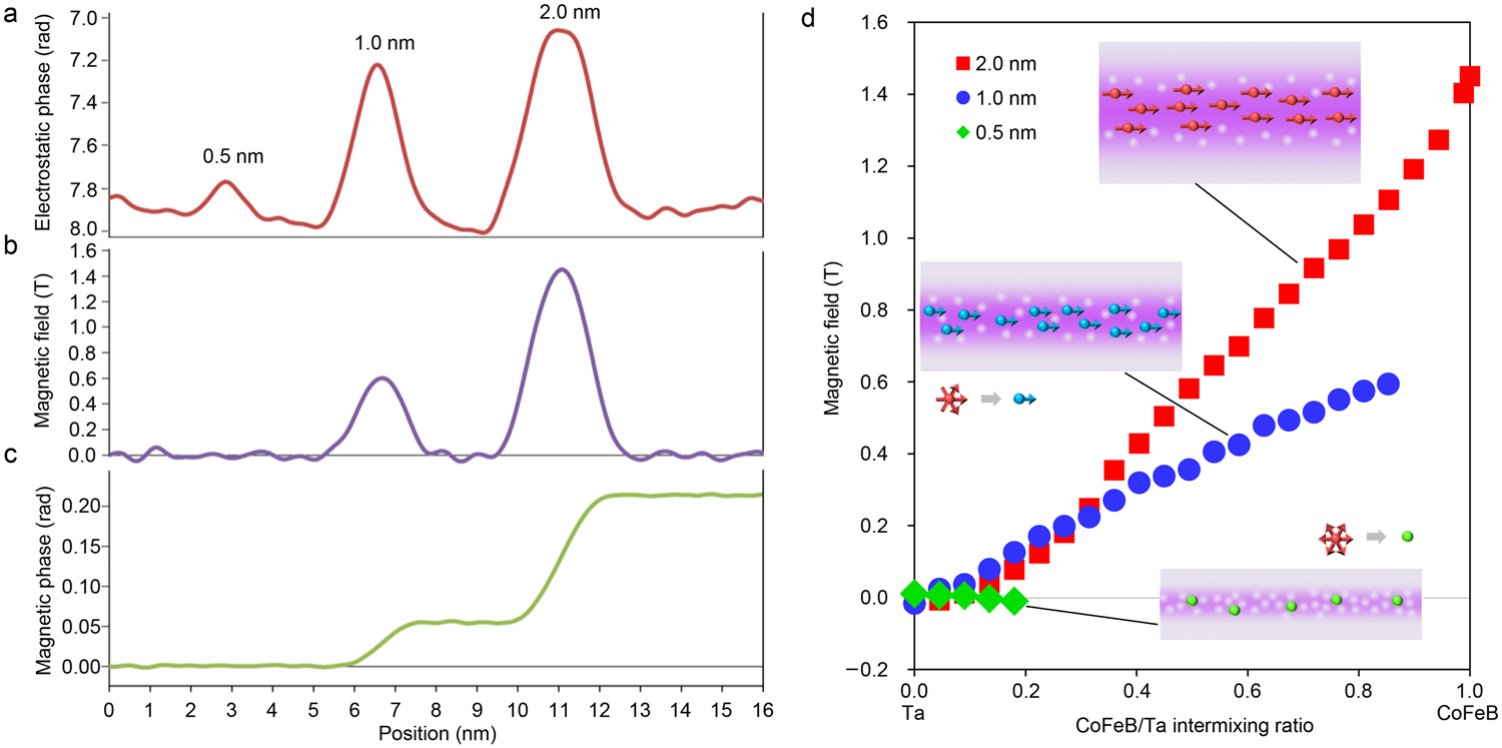
Magnetic field reduction by intermixing in CoFeB/Ta multilayer. Line profiles of electrostatic phase (**a**) magnetic field (**b**) and magnetic phase (**c**) of the same region of interest. Note that electrostatic phase is drawn reversely to magnetic phase. (**d**) Magnetic field as a function of the CoFeB/Ta intermixing ratio. Ferromagnetic order is stable in 2.0-nm-thick layer. The spin directions fluctuate in 1.0-nm-thick layer because intermixing of Ta atoms into the centre of the CoFeB layer causes disconnections of spin interactions. The highly mixed state of the 0.5-nm-thick layer results in the superparamagnetic state.

To compare the positions of the CoFeB layers and the magnetic field distributions in the area indicated by the black dashed rectangle in Fig. 3b, the profiles of the electrostatic phase (Fig. 5a) are shown alongside the magnetic field (Fig. 5b) obtained from the magnetic phase (Fig. 5c). Note that the electrostatic phase is flipped upside down to facilitate comparison with the magnetic field. The magnetic field at the centre of the 2.0-nm-thick CoFeB layer is 1.45 T. This is comparable to the result (1.50 T) macroscopically measured by a vibrating sample magnetometer (VSM). The magnetic field reduction in the intermixing area is observed over the magnetic field noise of 0.02 T (standard deviation of the profile in the Ta area). The magnetic field sensitivity in the line profile is made 0.06 T by setting the signal-to-noise ratio to 3. Although magnetic field changes smaller than 0.06 T cannot be detected in this measurement, changes larger than 0.06 T can be observed at 0.67-nm resolution. The peak of the magnetic field in the 1.0-nm-thick layer is lower than that in the 2.0-nm-thick layer. The distance from the centre of the 1.0-nm-thick layer to the interface may not seem to be large enough compared to the observed spatial resolution of 0.67 nm, however, holography simulation with the multislice method^31^ shows that quantitative measurement can be performed in the 1.0-nm-thick layer because the magnetic field distribution there changes gradually (Supplementary Information). Moreover, one advantage of electron holography is that the total amount of the magnetic flux in the layers can be detected by the size of the phase step across the layers due to the Aharonov-Bohm effect^32^. One sees in the magnetic phase profile (Fig. 5c) that the phase step is 0.0550 ± 0.0013 rad in the 1.0-nm-thick layer and 0.1604 ± 0.0013 rad in the 2.0-nm-thick layer. The magnetic fluxes derived from the phase steps in the 1.0-nm and 2.0-nm layers are 3.62 ± 0.09 × 10^−17^ Wb and 10.56 ± 0.09 × 10^−17^ Wb, respectively. The average magnetic phase gradient of 0.0550 ± 0.0013 rad/nm in the 1.0-nm layer is less than the gradient of 0.0802 ± 0.0007 rad/nm in the 2.0-nm-thick layer. This means that the average in-plane magnetic field in the 1.0-nm-thick layer is intrinsically smaller than that in the 2.0-nm-thick layer. Similar considerations are also applicable to the 0.5-nm-thick layer, where the phase step is 0.0001 ± 0.0013 rad (0.01 ± 0.09 × 10^−17^ Wb). The absence of the magnetic field in the 0.5-nm-thick layer is intrinsic and agrees with the fact that the spontaneous magnetization is not discerned in the SQUID measurements of the in-plane magnetization (Supplementary Information). The authors of a previous study^33^ have reported that CoFeB layers less than 0.9 nm thick show superparamagnetic-like behaviour. We therefore deduce that the unrecognized magnetic field in the 0.5-nm-thick layer in the present results is a consequence of superparamagnetism.

Another advantage of electron holography is that the composition ratio of CoFeB to Ta can be estimated from the electrostatic phase distribution (Fig. 5a) by using non-binding approximation^34^. Here we use linear relation for the electrostatic phase and composition ratio of CoFeB to Ta by assuming that the peak of the electrostatic phase in the 2.0-nm-thick layer comes from pure CoFeB and that the background electrostatic phase in Ta comes from pure Ta. The data in the plots of in-plane magnetic field versus the CoFeB/Ta intermixing ratio (Fig. 5d) are derived from the results shown in Fig. 5a and b. The interesting finding is that the spin ordering in the layer is individually featured by the degree of the CoFeB/Ta intermixing ratio for each layer. The magnetic field in the 2.0-nm-thick layer is characterized to be in accord with the bulk saturation magnetization of 1.5 T and gradually decreases as the Ta composition increases. On the other hand, the magnetic field in the 1.0-nm-thick layer is explicitly smaller than that in the 2.0-nm-thick layer at the same CoFeB/Ta intermixing ratio. In the 0.5-nm-thick layer, much intermixing occurs and the CoFeB compositions become quite low. Therein, the spontaneous magnetization cannot be discerned; the moment directions may fluctuate. The reduction of magnetic field in the 1.0-nm-thick layer is attributed to the starting of spin fluctuations due to disconnections of ferromagnetic spin interactions by Ta atoms at the centre of the layers. Like this, the high-resolution direct observation of magnetic field is powerful for this kind of amorphous materials, for which theoretical calculations of the band structure including spin density are difficult. The observed magnetic fields in the CoFeB/Ta multilayer indicate that the degree of intermixing of the heavy metal in the CoFeB layer is a key issue for controlling the spin ordering in spintronics devices.

In conclusion, the highest-resolution magnetic field observation in electron holography has been achieved by an aberration-corrected 1.2-MV holography electron microscope using a pulse magnetization system. The magnetic field in a sample CoFeB/Ta multilayer has been observed with 0.67-nm spatial resolution. The effect of intermixing at CoFeB/Ta interface on the magnetization has been analyzed by simultaneous observations of magnetic field and electrostatic potential distributions. The 1.2-MV holography electron microscope for exploring magnetic characteristics with high resolution can be used for various kinds of fundamental research and for developing practical applications and industrial devices.

## Methods

Magnetic multilayers of Ta(5.0 nm)/CoFeB(0.5 nm)/Ta(3.0 nm)/CoFeB(1.0 nm)/Ta(3.0 nm)/CoFeB(2.0 nm)/Ta(6.0 nm) were prepared on thermally oxidized Si substrates by sputtering deposition. The surface roughness *R*_a_ of the thermally oxidized Si was 0.1 nm. To evaluate the macroscopic magnetic properties of each magnetic layer, we deposited CoFeB samples with three different thicknesses (0.5, 1.0, and 2.0 nm) under the same conditions used to prepare the multilayer samples and measured the *M*-*H* curves of three samples with the magnetic field applied parallel (in-plane) and perpendicular (out-of-plane) to the film plane by using a vibrating sample magnetometer. For the in-plane magnetization, SQUID measurements were performed (Supplementary Information).

A thin sample was prepared by FIB milling (FIB-SEM NB5000, Hitachi High-Technologies Co.) at an acceleration voltage of 40 kV and Ar ion beam milling (PIPS Model 691, Gatan Inc.) at an acceleration voltage of 2.7 kV to remove the surface damaged layer. TEM observations were performed using a 300-kV TEM (HF-3300, Hitachi High-Technologies Co.).

Electron holograms with a fringe spacing of 0.22 nm were formed by double-biprism interferometry using an aberration-corrected 1.2-MV holography electron microscope^19,20^ (Hitachi, Ltd.) (Supplementary Information). The beam damage was evaluated by measuring the changes of the electrostatic and magnetic phases of the 2.0-nm-thick CoFeB layer from the first data to the last data: the electrostatic phase decreased 0.8 ± 0.1% and the magnetic phase decreased 1.5 ± 4.0%. Holograms were recorded using a direct electron detection camera (K2 Summit, Gatan Inc.). The holograms were reconstructed using HoloWorks v5.0, a plug-in for the Gatan Microscope Suite (GMS) v2.3 (Gatan Inc.). The aperture used in the reconstruction was set to allow spatial information greater than 0.66 nm to pass through. Self-coded scripts for GMS were used to process automatically acquired holograms. The magnetic fields were obtained from the magnetic phase profile by using equation (2) and the sample thickness of 45 nm.

## Electronic supplementary material

Supplementary Information
