# Supplementary material for: Magnetic field observations in CoFeB/Ta layers with 0.67-nm resolution by electron holography
Source: Sci Rep. 2017 Dec 5;7:16598. doi: 10.1038/s41598-017-16519-7 (PMC5717169; doi:10.1038/s41598-017-16519-7)
Supplement: Supplementary file 1 — Supplementary Information [file 41598_2017_16519_MOESM1_ESM.pdf]

SUPPLEMENTARY INFORMATION

Magnetic field observations in CoFeB/Ta layers with 0.67-nm resolution by electron holography

Toshiaki Tanigaki, Tetsuya Akashi, Akira Sugawara, Katsuya Miura, Jun Hayakawa, Kodai Niitsu, Takeshi Sato, Xiuzhen Yu, Yasuhide Tomioka, Ken Harada, Daisuke Shindo, Yoshinori Tokura and Hiroyuki Shinada

Table of Contents

1. Pulse magnetization system 2
2. Magnetization reversal in thin oxide sample 4
3. High-sensitivity magnetic phase measurements in thin oxide sample 5
4. Holography analysis for subnanometre-resolution observation 6
5. SQUID measurement results of CoFeB layers 8
6. Evaluation of quantitativeness in magnetic field observations through the simulation 8
References 11

1. Pulse magnetization system

The achievable resolution in magnetic field observation using electron holography is determined by the spatial resolution of the transmission electron microscope and the holography procedures used. The recent development of an aberration-corrected 1.2-MV holography electron microscope[1] enables a spatial resolution of 0.24 nm to be obtained at a magnetic-field-free sample position[2]. The remaining obstacle to achieving subnanometre-resolution magnetic field observation was separating the electrostatic and magnetic phases at high resolution. To overcome this obstacle, we developed a pulse magnetization system (Fig. S1). Using this system, we can reverse the sample's magnetization without mechanical movement and thereby separate the electrostatic and magnetic phases at high resolution.

The coils generating the magnetic fields are placed near the sample holder. The system has a side-entry sample holder, which facilitates various experimental setups, such as double tilting, heating, cooling, probing, and light illumination. A pulse current is used to reduce Joule heating of the coils. However, electromagnetic simulation using ELF/MAGIC 4.0 code (ELF Co., Japan) explained that an eddy current generated in the sample holder cancels the applied magnetic field if the pulse time width is less than 100 μs. We thus designed the pulse power supply to the increase the current at about 1 A/μs and cut it off when it reaches a set value. Figure S2 shows the measured profile of the pulse currents. The pulse width increases with the pulse amplitude.

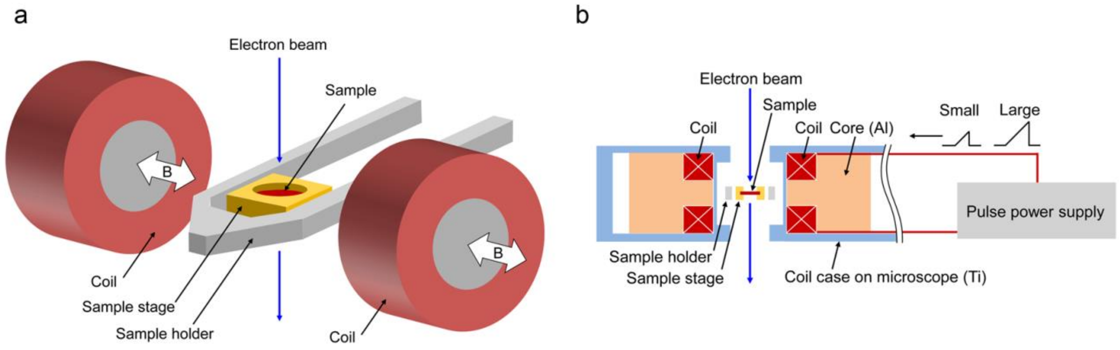

Figure S1 | Developed pulse magnetization system.

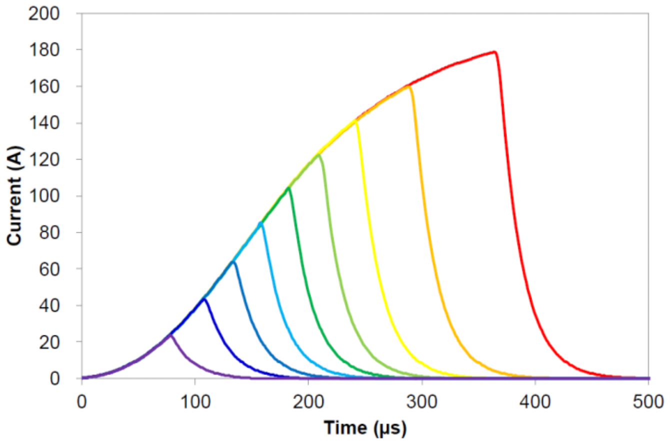

Figure S2 | Profiles of pulse currents applied to coil. Peak current can be flexibly controlled by adjusting power supply. Peak current was 180 A.

Magnetic field simulation using the measured pulse current was used to estimate the magnetic field at the sample position. The model used for the simulation (Fig. S3) was symmetrical around an axis (black dash-dotted line). The materials used in the simulations were the same as those used in the experiments.

Figure S4a shows the time-dependent magnetic field at the sample position for a pulse amplitude of 180 A with two types of holders (double-tilt and cryo holders) and without a holder. The double-tilt holder has a Ti sample stage part near the sample and a phosphor–bronze sample holder. The cryo holder has a Cu sample stage part near the sample and an SUS sample holder. With the double-tilt holder, an applied magnetic field at the sample can be almost same as that without a holder. With the cryo holder, the applied magnetic field should be lower, but the peak simulated magnetic field was 399 kA/m.

Figure S4b shows the magnetic field plotted against the pulse current measured with a Hall effect sensor and the simulated magnetic fields for the two types of holders and without a holder. Direct measurement at the sample position with the holders could not be performed due to the geometrical limitations of the sensors and holders. Therefore, the simulated values were used to estimate the magnetic field values at the sample position. Those estimated without the holder were the same as the measured values. The simulated magnetic field at the sample position reached 415 kA/m for the double-tilt holder and 399 kA/m for the cryo holder.

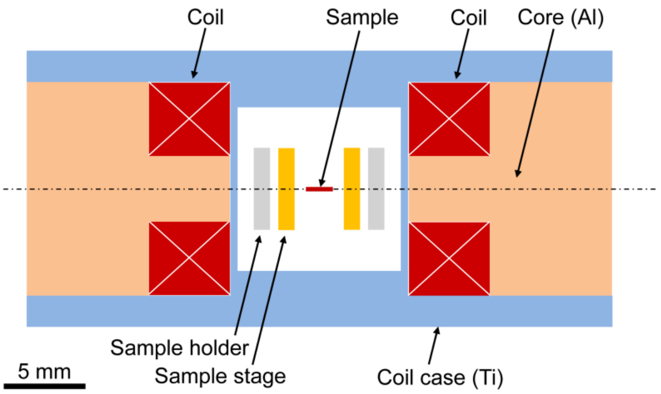

Figure S3 | Model used for simulation.

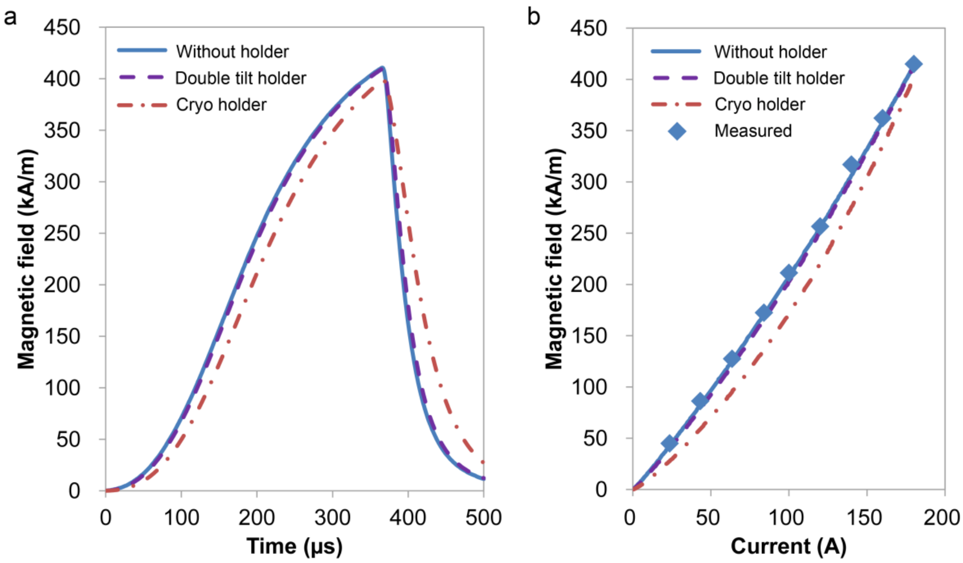

Figure S4 | Simulated and measured magnetic field at sample position with and without two types of sample holder. a, Time-dependent magnetic field for maximum pulse current of 180 A. **b**, Magnetic field vs. applied current. Magnetic field at sample position was measured using a Hall effect sensor.

2. Magnetization reversal in thin oxide sample

Magnetization reversal was investigated using a thin oxide magnetic sample (Ba_2FeMoO_6)[3]. The sample was prepared using focused ion beam (FIB) milling at an acceleration voltage of 40 kV and a micromanipulator and was finally subjected to Ar ion beam milling at an acceleration voltage of 500 V to remove the surface damaged layer. To confirm magnetization reversal in the sample, pairs of holograms were acquired by flipping the sample mechanically before and after applying the pulse magnetic field, and for each condition the electrostatic phases were subtracted (Fig. S5). The images in Fig. S5b and S5c clearly show reversal of the magnetization direction. The applied pulse magnetization field was 207 kA/m. Note that a pulsed magnetization field of less than 72 kA/m, which is maximum applicable magnetic field in the previous magnetization system for in-situ observations, was not strong enough to reverse the magnetization direction in this sample. These results show that the developed system can be applied for measuring the magnetic field in a sample whereas the previous magnetization system for the in-situ cannot be applied.

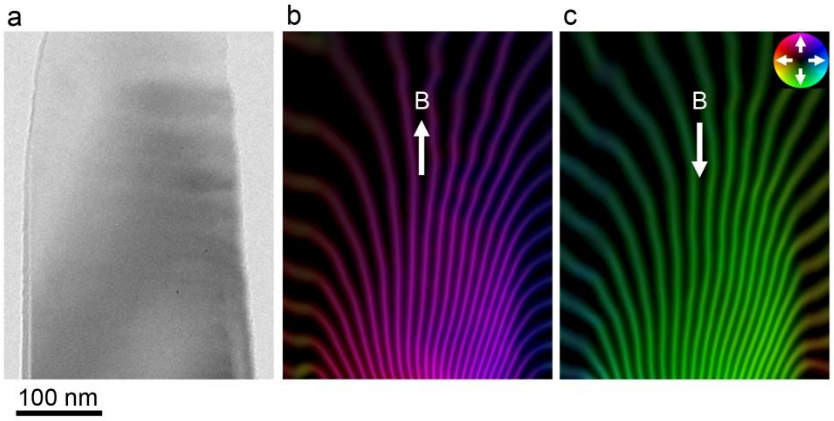

Figure S5 | Magnetization reversal in Ba_2FeMoO_6 using pulse magnetization system. a, TEM image; **b** and **c** magnetic flux distributions before and after magnetization reversal. Color wheel shows directions of magnetic flux, which is displayed by cosine of phase φ_M amplified 30 times ($\cos 30\varphi_M$). A constant flux of $h/30e$ flows between adjacent contour lines.

3. High-sensitivity magnetic phase measurements in thin oxide sample

To enable detection of a small phase shift by averaging out multiple phases[4-6], we developed an automated hologram acquisition system with a system for controlling the pulse magnetization system and camera. This system automatically acquires multiple sets of the holograms obtained before and after magnetization reversal. It was used to observe the magnetic phase in a thin Ba_2FeMoO_6 sample with uniform magnetization at a magnification of 75,000. There were 140 hologram sets, and the exposure time for each hologram was 10 s.

The acquired holograms were reconstructed with 8-nm resolution, and the magnetic phase shift was obtained (Fig. S6). The phase shown in Fig. S6a is amplified 100 times, and the color key shows the magnetization direction. Figure S6b shows the phase line profile for the white dashed rectangular area in Fig. S6a. The phase decreased linearly. This indicates that the observed area was a single domain with uniform magnetization. By assuming that the sample thickness is constant and the magnetization is uniform, we can evaluate the phase noise of the obtained magnetic phase by taking the differences from the linear fit of the obtained phase profile (Fig. S6c), which had a standard deviation of ±0.0021 (2π/2990) rad. The obtained phase noise was made to correspond to the phase resolution of 2π/1000-rad order by setting the signal-to-noise ratio to 3. These results show that the automated hologram acquisition system can be used to increase the signal-to-noise ratio and thereby achieve high-sensitivity magnetic phase measurement.

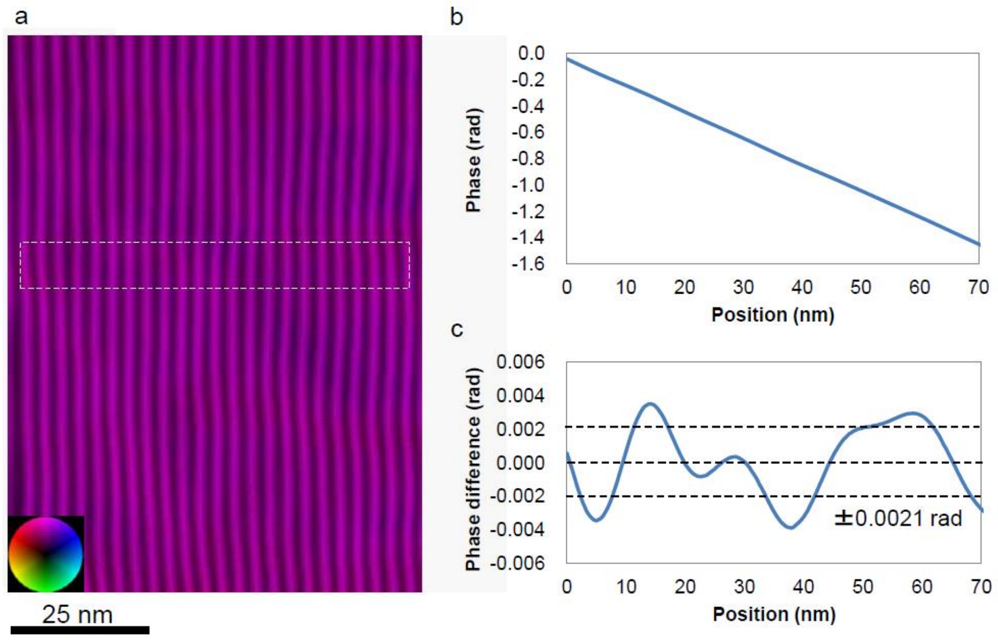

Figure S6 | Magnetic phase of single-domain Ba_2FeMoO_6 thin sample and its phase resolution obtained using pulse magnetization system. a, Magnetic flux displayed by cosine of phase φ_M amplified 100 times ($\cos 100\varphi_M$). **b**, Line profile of phase along dotted area indicated in **a**. **c**, Phase difference from linear phase profile, which is expected under the uniform magnetization for constant thickness. Phase noise in the observation evaluated by standard deviation of the phase difference was ±0.0021 (2π/2990) rad. The obtained phase noise was made to correspond to the phase resolution of 2π/1000-rad order by setting the signal-to-noise ratio to 3.

4. Holography analysis for subnanometre-resolution observation

High-resolution magnetic field observation by electron holography needs markers for position alignment to average out reconstructed phases and subtract a set of the phases. Platinum nanoparticles about 2 nm in diameter were deposited for this purpose by using a sputtering coater. A thin sample was prepared by FIB milling (FIB-SEM NB5000, Hitachi High-Technologies Co.) at an acceleration voltage of 40 kV. Reference areas near the observation areas were prepared by removing the surface carbon layer with a plasma cleaner. After the plasma procedure, the sample was thinned by Ar ion beam milling (PIPS Model 691, Gatan Inc.) at an acceleration voltage of 2.7 kV to remove the surface damaged layer. The sample thickness was monitored during the sample preparation by SEM. The CoFeB thickness of the sample was determined by cross-sectional TEM observation after holography observation to be 45 nm (Fig. S7). The width of the thin sample along the CoFeB multilayer was 1500 nm and the demagnetization field in a magnetic layer was small due to its wire-like shape.

The spatial resolution of the 1.2-MV holography electron microscope is 0.24 nm when the sample is located at a magnetic-field-free position. The achievable spatial resolution in magnetic field observation is greater than 0.24 nm and is determined by the hologram fringe spacing and reconstruction conditions. The reconstructed spatial resolution in electron holography is from two to three times the hologram fringe spacing, depending on the reconstruction conditions. Thus, if we want to obtain subnanometre spatial resolution, the hologram fringe spacing has to be less than 0.5 nm. In the experiment using the CoFeB multilayer, we set the hologram fringe spacing to 0.22 nm, as shown in Fig. S8. Holograms were formed by using double-biprism interferometry[7] and obtained by using a direct electron detection camera (K2 Summit, Gatan Inc.). The hologram fringe contrasts in the vacuum area used as the reference hologram were 40%. The aperture used in the reconstruction of the hologram was set to allow spatial information greater than 0.66 nm to pass through. A pulsed magnetic field of 207 kA/m was used to reverse the sample magnetization. The double-tilt sample holder was used for this multilayer sample to align the magnetic layer parallel to the electron beam. The exposure time for a hologram was 2 s, and 180 sets of holograms were acquired. Note that we applied the pulsed magnetic field parallel to the CoFeB multilayers, so only the ferromagnetic components pinned in the in-plane direction were finally visible.

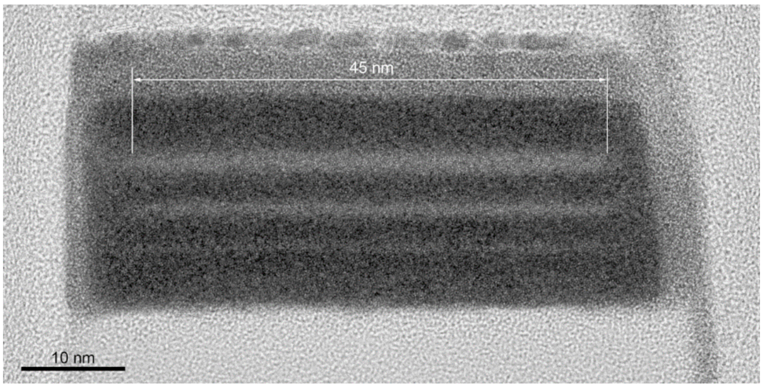

Figure S7 | Cross-sectional TEM image of thin sample. The CoFeB thickness, 45 nm, was determined by excluding the regions of CoFeB and Ta mixing at the sample surfaces.

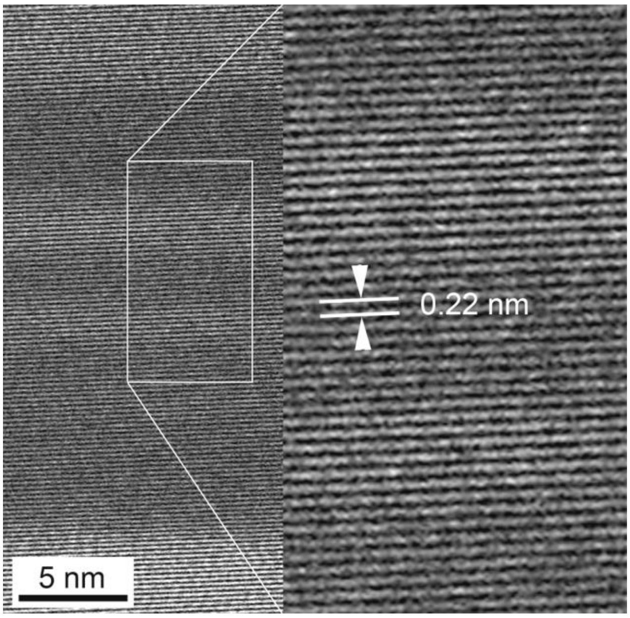

Figure S8 | Typical hologram for observing magnetic fields in CoFeB layers. Fringe spacing was 0.22 nm. Phase reconstruction was performed using an aperture that allowed spatial information greater than 0.66 nm to pass through.

5. SQUID measurement results of CoFeB layers

The in-plane magnetic properties of CoFeB layers measured by SQUID are shown in Fig. S9. The size of the sample were 5 mm×5 mm. 2.0-nm-thick and 1.0-nm-thick CoFeB layers showed spontaneous magnetization, but a 0.5-nm-thick layer did not.

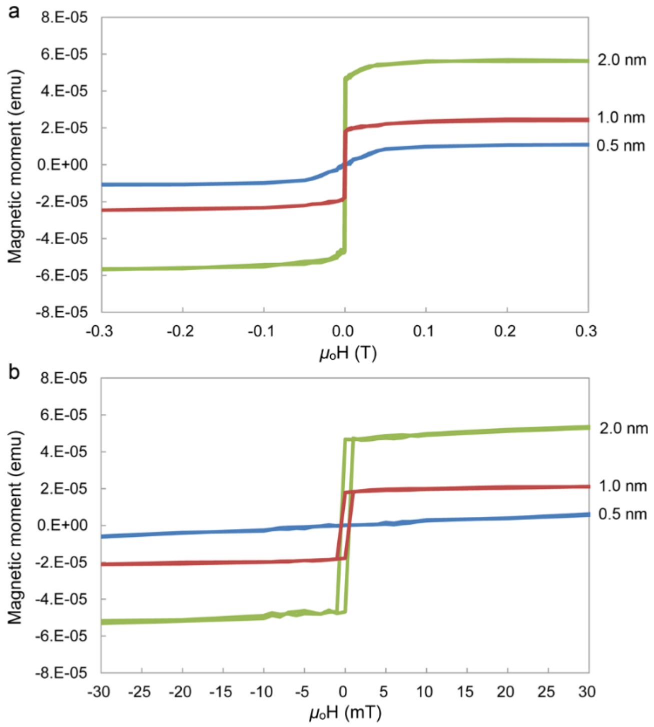

Figure S9 | SQUID measurement results of CoFeB layers. b is an enlargement of the low-field part of **a**.

6. Evaluation of quantitativeness in magnetic field observations through the simulation

To estimate the effect of the multi scattering in the sample and the holography observation, we carried out multislice simulation[8] including the lens effects and the effect of the reconstruction aperture used in the hologram reconstruction.

The one-dimensional model structure used in the simulation was a CoFeB(0.5 nm)/Ta(3.0 nm)/CoFeB(1.0 nm)/Ta(3.0 nm)/CoFeB(2.0 nm) multilayer sandwiched by Ta, and 0.35-nm Gaussian diffusion was assumed to represent the interface diffusion between CoFeB and Ta layer. The pixel size in the simulation was 0.05 nm/pixel, the slice thickness in the multislice simulation was 0.5 nm, and the beam convergence was not included. To compare the effects of the acceleration voltage of the microscope and the lens aberrations, simulations were performed for the 200-kV and 1.2-MV TEM. The magnetic field was set to be linear proportion to the composition ratio of CoFeB at Ta. The saturation magnetic field of the CoFeB was set to 1.5 T. The mean inner potentials of CoFeB and Ta were set to 24.1 V and

27.4 V by using the experimental results. The interaction constant C_E in the phase shift due to electrostatic potential for the 1.2-MV TEM is 5.31×10^{-3} radV^{-1}nm^{-1} and the C_E for the 200-kV TEM is 7.29×10^{-3} radV^{-1}nm^{-1}. The intensity attenuation[9] caused by the electron wave's passage through the sample was included by using the equation $I(t)=I_0\exp(-\mu t)$, where I is the wave intensity after passing through the sample, I_0 is the incident wave intensity, μ is the attenuation coefficient (which depends on the sample and objective aperture size), and t is the sample thickness. The coefficients μ_{Ta} and μ_{CoFeB} for the 1.2-MV TEM were set to be 0.01604 nm^{-1} and 0.00997 nm^{-1} by using the observation results. The coefficients μ_{Ta} and μ_{CoFeB} for the 200-kV were calculated from the values for 1.2-MV TEM by using the ratio of 1.89, which is the ratio of the square of the velocity of a 200-keV electron wave to that of a 1.2-MeV electron wave[10]. The effect of the reconstruction aperture was applied in the FFT space in the way it was in the hologram reconstruction and set so as to allow spatial information greater than 0.66 nm to pass through.

The parameters for the 200-kV TEM were the following: defocus = 0 nm (Gaussian focus) and spherical aberration C_3 = 0.6 m. The sample thickness was set to 50 nm. The results are shown in Figure S10. Note that the profile of the image plane is overlapped on the holography results. For the case with a large spherical aberration coefficient, the fringing effect due to the lens aberration is the main reason for artifacts in the magnetic field analysis.

The parameters for the 1.2-MV TEM were obtained experimentally and set to the following values: defocus = 40 nm (to minimize the phase shift in the reciprocal space and the TEM phase contrast), C_3 = -49 mm, and fifth-order spherical aberration C_5 = 2140 m. The results for the sample thickness of 50 nm are shown in Figure S11(a–d). Note that the profile of the image plane is overlapped on the holography results. The magnetic fields are simulated for 50 nm, 100 nm, and 150 nm (Fig. S11e). The multiple-scattering effect in the sample is negligibly small in a sample less than 50 nm thick (Fig. S11f).

The intensity of a 1.2-MV TEM is larger than that of a 200-kV TEM. The phase shift in the sample as well as the fringing effect caused by the defocus and aberration in a 1.2-MV TEM is smaller than that caused by the defocus and aberration is smaller in the 1.2-MV TEM than in the 200-kV TEM.

To evaluate the quantitativeness in the magnetic field observations by the 1.2-MV TEM for the 1.0-nm-thick layer, we performed multislice simulation for three models of magnetic field distributions (Fig. S11g). The distribution in Model 1 was set to be similar to that observed in the 1.0-nm-thick CoFeB layer. The distributions in Model 2 and Model 3 were set to be narrower than that in Model 1. Note that the integral values were set to be the same in all models. The pixel size in this simulation was 0.03592 nm/pixel, and the sample thickness was set to 50 nm. The other parameters were the same as those used in the above calculation. The shot noise of electrons was omitted in order to distinguish the limiting factors of hologram reconstruction from the signal-to-noise ratio in the quantitative measurements.

The simulated magnetic field shows profiles almost the same as the three models of magnetic field distributions. The simulation results indicate that the observation condition is good enough to evaluate magnetic field distribution in the 1.0-nm layer in this experiment. The results can be interpreted as following: The CoFeB/Ta multilayer shows intermixing at the interfaces and the composition distribution changes gradually between Ta and CoFeB regions. This gradual composition change leads to the gradual change of magnetic field distribution and it can be mainly expressed by spatial frequencies within 1/0.66 nm^{-1}.

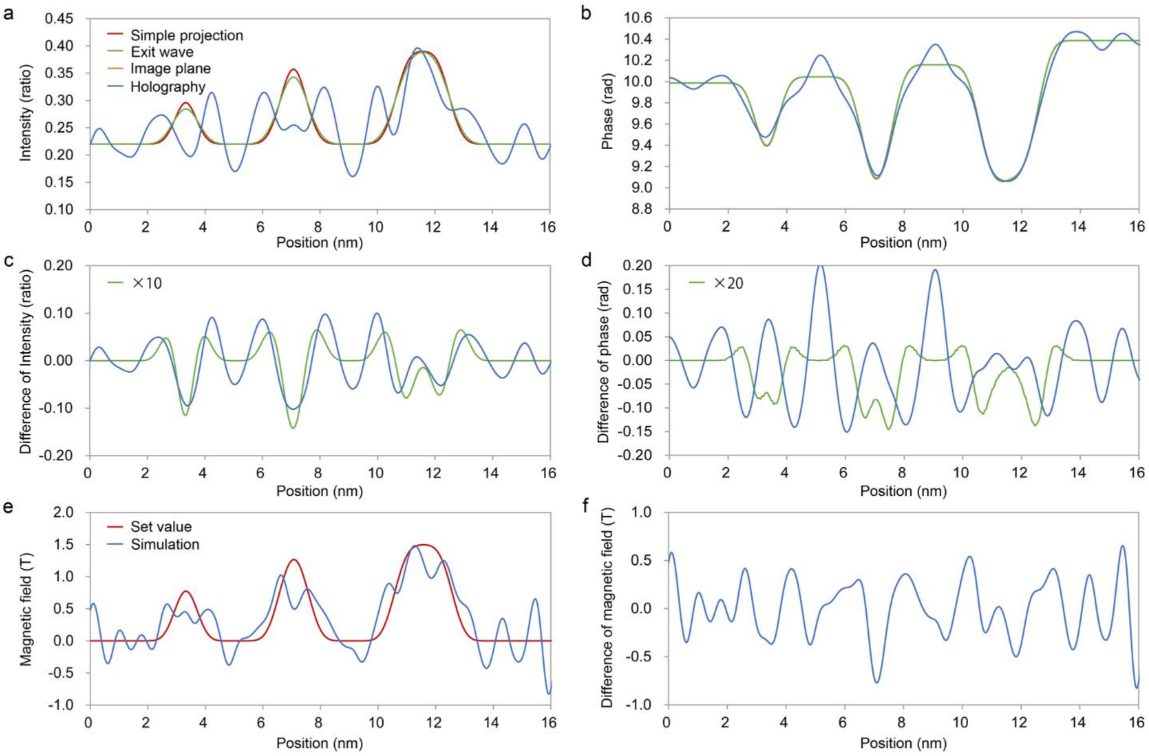

Figure S10 | The multislice simulation results for 200-kV TEM. a, Intensity. **b**, Phase. **c**, Difference of intensity from the model (simple projection). **d**, Difference of phase from the model. **e**, Magnetic field. **f**, Difference of magnetic field from the set value.

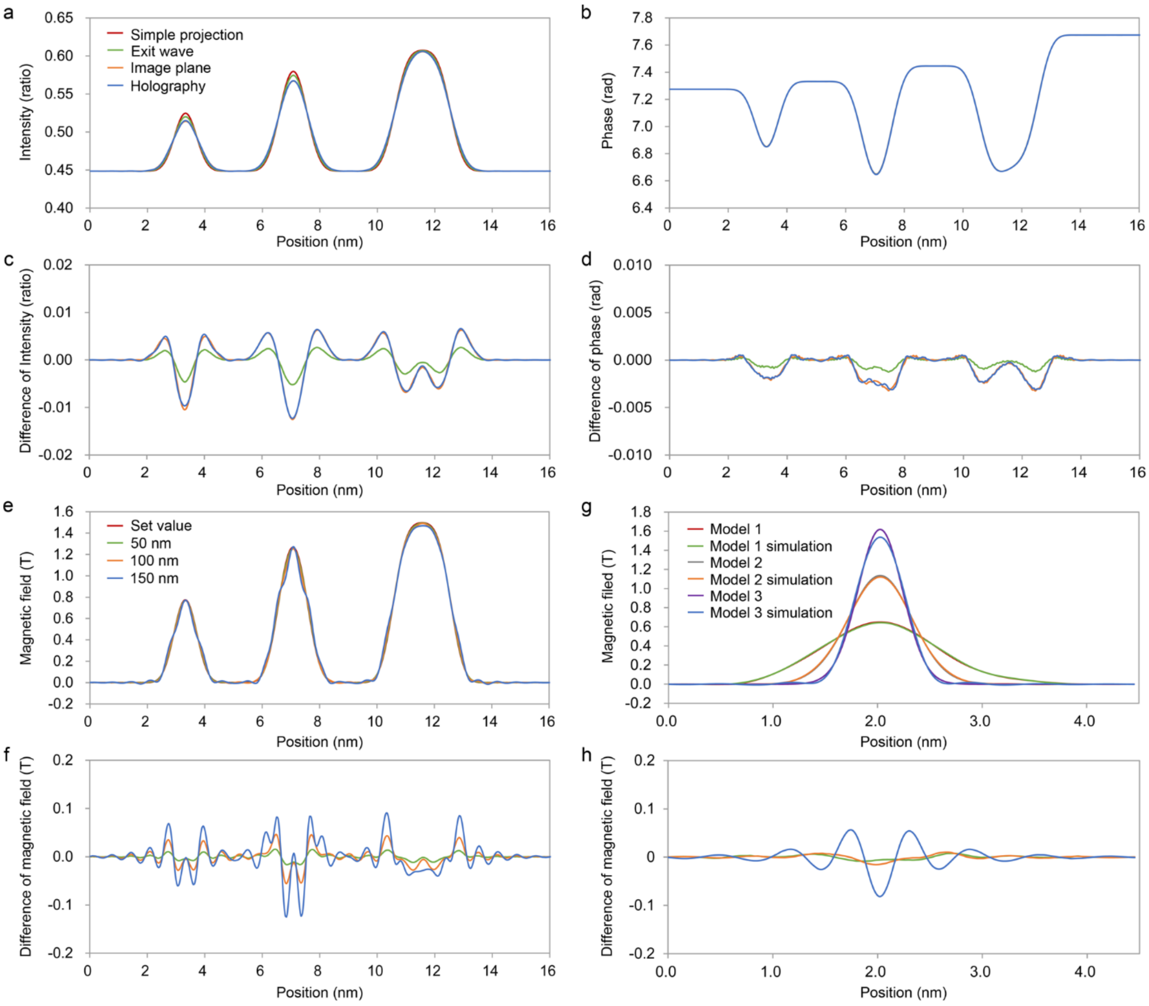

Figure S11 | The multislice simulation results for 1.2-MV TEM. a, Intensity. **b**, Phase. **c**, Difference of intensity from the model (simple projection). **d**, Difference of phase from the model. **e**, Magnetic field. **f**, Difference of magnetic field from the set value. **g**, Three models of magnetic field distributions and simulation results. **h**, Differences of simulated magnetic fields from the models.

<u>References</u>

1. Akashi, T. *et al.* Aberration corrected 1.2-MV cold field-emission transmission electron microscope with a sub-50-pm resolution. *Appl. Phys. Lett.* **106,** 074101 (2015).
2. Tanigaki, T., Akashi, T., Takahashi, Y., Kawasaki, T. & Shinada, H. Quest for ultimate resolution using coherent electron waves: An aberration-corrected high-voltage electron microscope. *Adv. Imaging Electron Phys.* **198,** 69-125 (2016).
3. Tomioka, Y. *et al.* Magnetic and electronic properties of a single crystal of ordered double perovskite Sr_2FeMoO_6. *Phys. Rev. B* **61,** 442-427 (2000).

4. Yamamoto, K., Kawajiri, I., Tanji, T., Hinbino, M. & Hirayama, T. High precision phase-shifting electron holography. *J. Electron Microsc.* **49,** 31-39 (2000).
5. Voelkl, E. & Tang, D. Approaching routine 2*π*/1000 phase resolution for off-axis type holography. *Ultramicroscopy* **110,** 447-459 (2010).
6. Suzuki, T. *et al.* Improvement of the accuracy of phase observation by modification of phase-shifting electron holography. *Ultramicroscopy* **118,** 21-25 (2012)
7. Harada, K., Tonomura, A., Togawa, Y., Akashi, T., & Matsuda, T. Double-biprism electron interferometry. *Appl. Phys. Lett.* **84,** 3229-3231 (2004).
8. Dunin-Borkowski, R. E., McCartney, M. R., Smith, D. J. & Parkin, S. S. P. Towards quantitative electron holography of magnetic thin films using in situ magnetization reversal. *Ultramicroscopy* **74,** 61-73 (1998).
9. Reimer, L. & Kohl, H. *Transmission electron microscopy*, 5th edition (Springer, New York, 2008).
10. Egerton, R. F. Choice of operating voltage for a transmission electron microscope. *Ultramicroscopy* **145,** 85-93 (2014).
